# Corallopyronin A for short-course anti-wolbachial, macrofilaricidal treatment of filarial infections

**DOI:** 10.1371/journal.pntd.0008930

**Published:** 2020-12-07

**Authors:** Andrea Schiefer, Marc P. Hübner, Anna Krome, Christine Lämmer, Alexandra Ehrens, Tilman Aden, Marianne Koschel, Helene Neufeld, Lillibeth Chaverra-Muñoz, Rolf Jansen, Stefan Kehraus, Gabriele M. König, Domen Pogorevc, Rolf Müller, Marc Stadler, Stephan Hüttel, Thomas Hesterkamp, Karl Wagner, Kenneth Pfarr, Achim Hoerauf

**Affiliations:** 1 Institute for Medical Microbiology, Immunology and Parasitology, University Hospital Bonn, Bonn, Germany; 2 German Center for Infection Research (DZIF), partner site Bonn-Cologne, Bonn, Germany; 3 Department of Pharmaceutical Technology and Biopharmaceutics, University of Bonn, Bonn, Germany; 4 Department Microbial Drugs, Helmholtz Center for Infection Research, Braunschweig, Germany; 5 German Center for Infection Research (DZIF), partner site Hannover-Braunschweig, Germany; 6 Institute for Pharmaceutical Biology, University of Bonn, Bonn, Germany; 7 Department Microbial Natural Products, Helmholtz Institute for Pharmaceutical Research Saarland, Saarbrücken, Germany; 8 Translational Project Management Office (TPMO), German Center for Infection Research, Braunschweig, Germany; University of Utah, UNITED STATES

## Abstract

Current efforts to eliminate the neglected tropical diseases onchocerciasis and lymphatic filariasis, caused by the filarial nematodes *Onchocerca volvulus* and *Wuchereria bancrofti* or *Brugia* spp., respectively, are hampered by lack of a short-course macrofilaricidal–adult-worm killing–treatment. Anti-wolbachial antibiotics, e.g. doxycycline, target the essential *Wolbachia* endosymbionts of filariae and are a safe prototype adult-worm-sterilizing and macrofilaricidal regimen, in contrast to standard treatments with ivermectin or diethylcarbamazine, which mainly target the microfilariae. However, treatment regimens of 4–5 weeks necessary for doxycycline and contraindications limit its use. Therefore, we tested the preclinical anti-*Wolbachia* drug candidate Corallopyronin A (CorA) for *in vivo* efficacy during initial and chronic filarial infections in the *Litomosoides sigmodontis* rodent model. CorA treatment for 14 days beginning immediately after infection cleared >90% of *Wolbachia* endosymbionts from filariae and prevented development into adult worms. CorA treatment of patently infected microfilaremic gerbils for 14 days with 30 mg/kg twice a day (BID) achieved a sustained reduction of >99% of *Wolbachia* endosymbionts from adult filariae and microfilariae, followed by complete inhibition of filarial embryogenesis resulting in clearance of microfilariae. Combined treatment of CorA and albendazole, a drug currently co-administered during mass drug administrations and previously shown to enhance efficacy of anti-*Wolbachia* drugs, achieved microfilarial clearance after 7 days of treatment at a lower BID dose of 10 mg/kg CorA, a Human Equivalent Dose of 1.4 mg/kg. Importantly, this combination led to a significant reduction in the adult worm burden, which has not yet been published with other anti-*Wolbachia* candidates tested in this model. In summary, CorA is a preclinical candidate for filariasis, which significantly reduces treatment times required to achieve sustained *Wolbachia* depletion, clearance of microfilariae, and inhibition of embryogenesis. In combination with albendazole, CorA is robustly macrofilaricidal after 7 days of treatment and fulfills the Target Product Profile for a macrofilaricidal drug.

## Author summary

Infections with filarial roundworms can cause the disfiguring human neglected tropical diseases onchocerciasis and lymphatic filariasis. Treatment of these diseases is limited, as there is no well-tolerated treatment available that kills the adult worms after a short-term regimen. Thus, mass drug administrations (MDA) are performed with drugs that temporarily clear the microfilariae, the filarial offspring, to inhibit the transmission of the disease. As these MDA treatments have to be given 1–2 times per year for many years, the goal to eliminate onchocerciasis and lymphatic filariasis is hampered. In the present study we investigated a novel preclinical candidate for the treatment of filariasis. Corallopyronin A (CorA) is a natural compound that clears the essential *Wolbachia* endobacteria of filariae. Using the *Litomosoides sigmodontis* rodent model of filariasis we demonstrated that 2 weeks of CorA treatment clears *Wolbachia* endosymbionts *in vivo*, leading to a maintained clearance of microfilariae by inhibition of filarial embryogenesis. Combination therapy of CorA with the MDA drug albendazole allowed lower CorA doses and shortened treatment to 7 days. More importantly, it also led to the death of the adult filariae. Portfolios (Target Product Profiles) of new drugs against filariae should show adult killing efficacy like CorA.

## Introduction

The new WHO roadmap 2030, referring to the United Nations Sustainable Development Goals, state that the Neglected Tropical Diseases onchocerciasis and lymphatic filariasis should be eliminated by 2030, at least in the majority of endemic countries [1,2]. Onchocerciasis and lymphatic filariasis are caused by filarial nematodes and represent a great public health and economic burden for endemic countries. Onchocerciasis can cause severe dermatitis and vision loss, including blindness, with 99% of cases occurring in sub-Saharan Africa. Lymphatic filariasis patients can suffer from lymphedema in limbs and the scrotum. Current efforts to eliminate both filarial diseases are hampered by the lack of a macrofilaricidal drug, i.e. killing the adult worms that will otherwise live for 5–15 years, continuously producing new offspring, the microfilariae (MF), thus sustaining the transmission cycle. Current control strategies are restricted to (semi-) annual mass drug administration (MDA) with drugs that temporarily inhibit embryogenesis and lead to clearance of the MF, with the aim to interrupt the transmission of the disease. However, the clearance of MF is not permanent and they rebound with levels suitable for transmission within a few months. In areas endemic for onchocerciasis, MDA with ivermectin or ivermectin plus albendazole (ALB) are given for both onchocerciasis and lymphatic filariasis [3], whereas in areas outside of Africa a triple therapy with ivermectin, ALB and diethylcarbamazine is recommended by the WHO for lymphatic filariasis [4].

The triple therapy is highly effective at producing long-term amicrofilaremia after one round of administration [5,6]. However, implementation of the triple therapy in Africa is hindered, as potential serious adverse events to diethylcarbamazine in onchocerciasis patients or people infected with the filarial nematode *Loa loa* prevent its use in most of the continent [7–10]. Therefore, with the current MDA treatment program for onchocerciasis, it is unlikely that the goal to eliminate onchocerciasis by 2030 will be met, as the current MDA treatments have to be given for the reproductive lifespan of *Onchocerca volvulus*, which exceeds 10 years [11–17].

An alternative and safe treatment regimen is the targeting of the *Wolbachia* spp. endosymbionts present in the majority of human pathogenic filariae (except *L*. *loa)* [18–20]. Using doxycycline it was shown for both onchocerciasis and lymphatic filariasis patients that this approach leads to permanent sterilization of the adult female filariae, clearance of MF and the slow death of the adult worms over months [21–23]. However, doxycycline cannot be given to pregnant and breast-feeding women nor to children below the age of 8, and its prolonged daily treatment of 4–5 weeks for onchocerciasis and lymphatic filariasis is not in accordance with an MDA approach. Nevertheless, doxycycline is a valuable tool for individual antifilarial therapy and is now recommended by the WHO for end-game strategies to eliminate onchocerciasis [24].

To achieve elimination of onchocerciasis and lymphatic filariasis, new drugs that provide a macrofilaricidal effect with 1–2 weeks of treatment are required with the aim to use them, if not for MDA, then more widespread than is possible with doxycycline. In the present study we used the *Litomosoides sigmodontis* rodent model of filariasis to assess the efficacy of the anti-*Wolbachia* candidate Corallopyronin A (CorA). CorA is a natural product of the myxobacteria *Corallococcus coralloides* that inhibits bacterial DNA-dependent RNA polymerase and is highly effective against Gram-positive bacteria, and Gram-negative bacteria that lack a *tolC* or other efflux pumps [25,26]. Although *Wolbachia* endosymbionts of filariae are Gram-negative, they have undergone significant genome reduction that has resulted in incomplete efflux pump pathways and inability to make lipopolysaccharide [27]. We have shown that *Wolbachia* are susceptible to CorA *in vitro* [28], and an *in vivo* 28-day CorA treatment of mice beginning one day after infection with *L*. *sigmodontis* depleted *Wolbachia* endosymbionts and inhibited the molting of infectious L3 into adult filariae [28].

Herein, we extended the previous results and evaluated the *in vivo* efficacy of CorA against the adult stage of *L*. *sigmodontis*, its impact on the *Wolbachia* endosymbionts, the MF load, embryogenesis and adult worm burden. For this we used the *L*. *sigmodontis* mouse and jird (Mongolian gerbils, *Meriones unguiculatus*) models, which had previously been selected by the Bill & Melinda Gates Foundation’s “Macrofilaricidal Drug Accelerator” program as suitable to evaluate the *in vivo* efficacy of leading drug candidates for filariasis [29–35]. The model in jirds harbors adult filariae for more than 1 year. Therefore, long-term effects of anti-filarial therapy can be assessed, e.g. macrofilaricidal activity [36]. Furthermore, we tested combinations with ALB that, although it has no substantial effect on *Wolbachia* on its own, was recently shown to synergize with anti-*Wolbachia* compounds, allowing shorter treatment regimens [37].

Our results demonstrate that 14-day CorA treatment is sufficient to reduce the *Wolbachia* burden by >99% in adult worms and MF, reduces the adult worm burden, inhibits embryogenesis in remaining worms, and clears the MF. In addition, a combination of CorA with ALB allows lower CorA doses and reduces the treatment duration to 7 days that results in significant macrofilaricidal efficacy.

## Methods

### Ethics statement

All animal experiments were conducted according to European Union Directive 2010/63/EU and were approved by the State Agency for Nature, Environment and Consumer Protection North Rhine-Westphalia, Germany (AZ 84–02.04.2015.A507). Animals were checked daily for food, water, and the weight and wellbeing of each animal was determined once a week. Severity of symptoms were assessed using a score of A-C that monitored weight loss, appearance and behavioral changes. Weight loss <10% following treatment and individual animals with minor wounds due to territorial behavior (both score A) were observed. No severe drug-caused symptoms of score B or C were recorded. For necropsies, animals were euthanized per inhalation of an overdose of isoflurane (AbbVie Deutschland GmbH & Co. KG, Wiesbaden, Germany).

### Animals

Female BALB/c mice (7–8 weeks old) were obtained from Janvier (Le Genest-Saint-Isle, France) and infected naturally as previously described [28]. Female Mongolian gerbils (*M*. *unguiculatus*; jirds; 7–10 weeks old) were obtained from Janvier or Charles River (Charles River Laboratories, Inc., Wilmington, MA, USA) and infected naturally as described [30,32]. Animals were housed in individually ventilated cages (Tecniplast, Buguggiate, Italy) at the animal facility of the Institute for Medical Microbiology, Immunology and Parasitology, University Hospital Bonn, Bonn, Germany. Animals were maintained on a 12 h light/dark cycle with constant access to food and water.

### Preparation of CorA

CorA was produced by the CRO Acies Bio (Ljubljana, Slovenia) using the natural producer strain *Corallococcus coralloides* B035 [28,38], or the Helmholtz Centre for Infection Research, Department of Microbial Drugs using the heterologous producer strain *Myxococcus xanthus* carrying the CorA biosynthesis gene cluster [39]. The CorA batches always exceeded a purity of 92%-96% as determined via ^1^H-NMR (S1 Fig). CorA was formulated in a 1:1 solution of polyethylene glycol 400 (PEG400; Carl Roth GmbH + Co. KG, Karlsruhe, Germany) and phosphate buffered saline, pH 7.4 (PBS; Gibco, ThermoFisher Scientific GmbH, Dreieich, Germany) for intraperitoneal (IP) administration. For oral (PO) administration, a formulation comprising CorA (20% weight), propylene carbonate (30% weight, ≥ 99.7%, Carl Roth GmbH + Co. KG), and Syloid XDP 3050 (50% weight, Grace GmbH, Worms, Germany) suspended in PBS was administered.

### Experimental design

Mice infected with *L*. *sigmodontis* were randomly assigned to the treatment groups and treatment was initiated the day after infection. Efficacy trials in mice were performed with IP treatments ranging from 72 to 2.5 mg/kg (1:2 dilution steps) CorA once a day (QD) for 7, 10 or 14 days. For PO efficacy, mice were administered 18 mg/kg CorA QD for 14 days. Both mouse study end points were 35 days post infection (dpi), a time at which *L*. *sigmodontis* have molted into adult worms in untreated mice. After euthanasia, if present, 10 female worms were collected and frozen at -20°C for quantification of *Wolbachia* by qPCR.

Jirds infected with *L*. *sigmodontis* were sampled 11 weeks post infection (wpi) to determine the presence of MF. MF positive jirds were randomly assigned to the treatment groups. In the first efficacy experiment, the infected animals were treated IP with CorA at 10 mg/kg twice per day (BID), or 30 mg/kg QD or BID for 14 days. In the second jird experiment, MF-positive jirds were treated BID with 30 mg/kg CorA IP for 14 days, or BID with 20 mg/kg CorA IP for 10 or 14 days. Corresponding controls received only vehicle treatment. In the third efficacy experiment, infected jirds were treated IP with 30 mg/kg CorA BID for 7, 10 or 14 days. In a fourth jird study, CorA was administered IP at 30 mg/kg or 10 mg/kg BID for 14 days, or as a combination of IP 10 mg/kg CorA BID plus 13 mg/kg ALB QD given PO for 7 days. CorA was formulated 1:1 in PEG 400:PBS, while ALB was administered as a suspension in 0.5% carboxymethylcellulose (Sigma-Aldrich Chemie, Taufkirchen, Germany), 0.5% benzyl alcohol (Carl Roth GmbH + Co. KG), 0.4% polysorbate 80 (Fagron GmbH & Co. KG, Glinde, Germany) and 0.9% NaCl (Fresenius Karbi Deutschland GmbH, Bad Homburg, Germany). Corresponding vehicle controls were used.

In all experiments with jirds, every other week starting 1 week before treatment onset, blood was drawn from the vena saphena for MF counts and determination of MF-*Wolbachia* loads by qPCR. The primary endpoint was defined as the long-term absence of MF for 18 weeks (jird experiment 1, 3, 4) or 21 weeks (jird experiment 2) after treatment onset.

For MF counts, 10 μL of blood were transferred to 190 μL of red blood cell (RBC) lysis buffer (BioLegend, Koblenz, Germany) and stored at 4°C until analysis. Ten microliters of the suspension were transferred to a slide and MF were counted using a microscope. If fewer than 10 MF were counted, the remaining suspension was centrifuged at 400 g for 5 min, the supernatant discarded and the pellet transferred to a slide to count the MF. Jirds were euthanized 16- or 18-weeks post treatment start (wpt) with an overdose of isoflurane (AbbVie Deutschland GmbH & Co. KG). The pleural cavity was opened and worms were collected and counted. Up to 10 female worms per animal were frozen at -20°C for quantification of *Wolbachia* with qPCR. Except for the combination therapy study, up to 5 female worms per jird were collected in 4% formaldehyde/PBS (Sigma Aldrich, St. Louis, MO, USA) for 24 h and then transferred into 60% ethanol for subsequent embryograms [33].

### *Wolbachia* quantification

DNA was extracted from MF and adult worms using QIAamp Mini kits (Qiagen, Hilden, Germany) as previously described [28,29]. *Wolbachia* quantification in adult female worms from mice and jirds was determined by qPCR using primers for the *Wolbachia* single copy gene *ftsZ* (GenBank Accession No.: AJ010271) and the *L*. *sigmodontis* actin gene (GenBank Accession No.: GU971367) for normalization as previously described [28]. The duplex real-time PCR was performed in triplicate using the primers (MicroSynth AG, Balgach, Switzerland) and hybridization probes (Biomers, Ulm, Germany): LsFtsZ FW 5’-cgatgagattatggaacatataa-3’, LsFtsZ RV 5’-ttgcaattactggtgctgc-3’, LsFtsZ TQP 5’-6-FAM-cagggatgggtggtggtactggaa-TAMRA-3’, LsActin FW 5’-atccaagctgtcctgtctct-3’, LsActin RV 5’-tgagaattgatttgagctaatg-3’, LsActin TQP 5’-HEX-actaccggtattgtgctcgatt-TAMRA-3’ in QuantiNova PCR mix (Qiagen). After activating the Taq at 95°C for 2 min, DNA was amplified in a Rotor-Gene Q (Qiagen) with 45 cycles of 95°C denaturing for 5 sec and 58°C annealing for 30 sec. Fluorescence was measured at the end of each annealing step. Standards for quantification used a mixed sample of plasmids containing the LsFtsZ and LsActin sequences.

To determine *Wolbachia* depletion in MF, 50 μL of peripheral blood from jirds was added to 950 μL RBC lysis buffer (Biolegend, San Diego, CA, USA) and stored at room temperature for 1 hour. After the incubation, samples were centrifuged for 5 min at 400 g, the supernatant was discarded, and the pellet suspended in 200 μL AE buffer (Qiagen). 50 MF, if present, were picked using a microscope and stored in AE-buffer at 4°C. The same qPCR was performed as described for the adult worms. The *ftsZ* copies/μL were normalized to actin copies/μL to control for size differences between worms that had molted to adults as opposed to those that were inhibited in development [40]. To clearly distinguish MF that were depleted of *Wolbachia* from the absence of MF due to successful inhibition of embryogenesis, we defined *ftsZ*/MF in blood samples from animals with no MF, but with an *ftsZ* signal above the detection limit (division by zero error), as 1X10^-4^.

### Embryograms

Embryograms were performed from female *L*. *sigmodontis* adult worms isolated from jirds at 18 or 21 wpt as previously described [32]. Briefly, single female adult worms were homogenized in 80 μL PBS. Then 20 μL Hinkelmann solution (0.5% eosin Y (Sigma-Aldrich Chemie), 0.5% phenol (Sigma-Aldrich Chemie), 0.185% formaldehyde (Sigma-Aldrich Chemie)) was added and mixed. Then the total volume was applied to a slide and the embryonic stages: eggs, morulae, pretzel, stretched MF, as well as early and late degenerated stages were counted.

### Statistics

**Fig 1:**
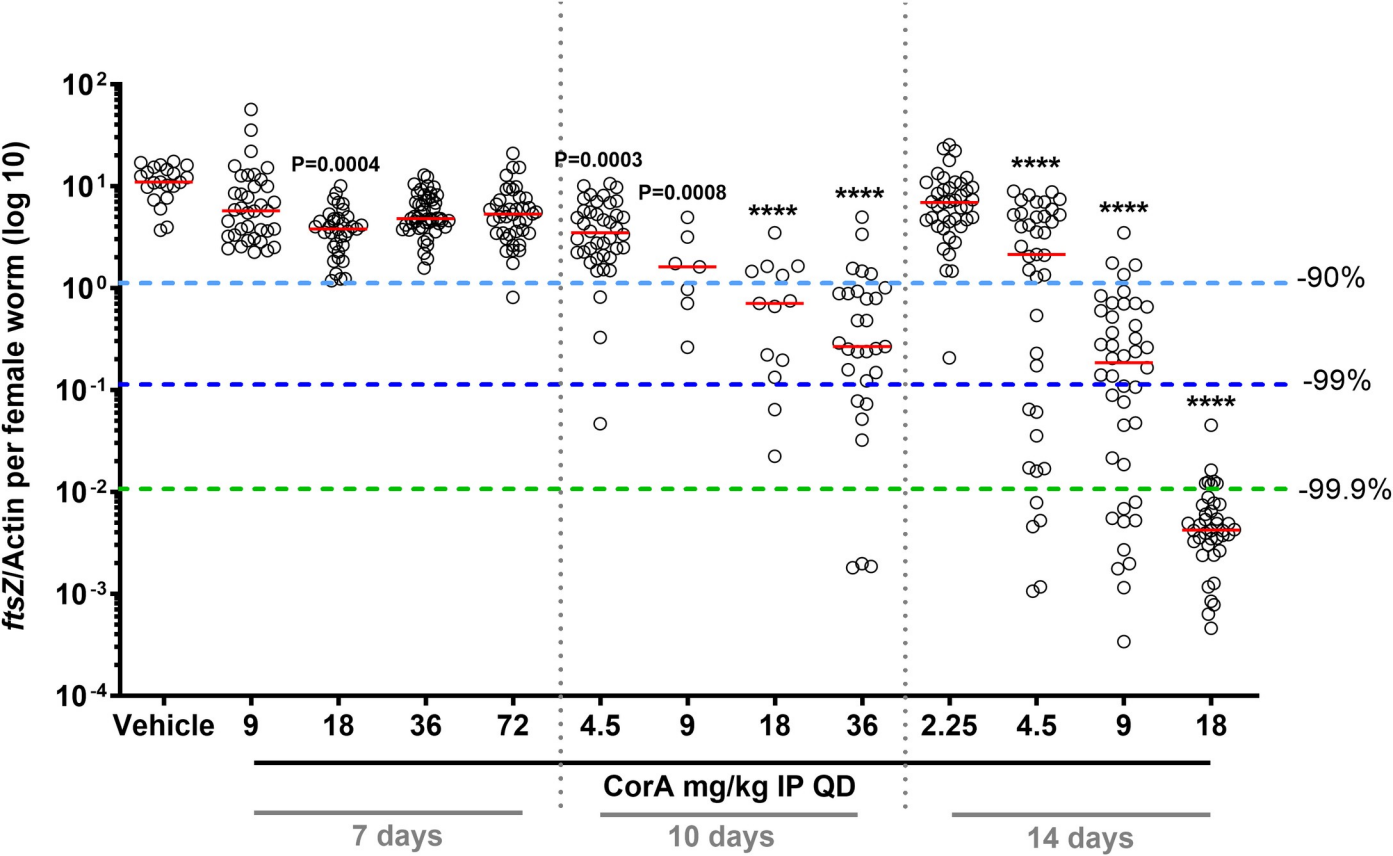
Fourteen-day CorA IP treatment depletes *Wolbachia* endosymbionts in the *L*. *sigmodontis* larval mouse model. One day after *L*. *sigmodontis* infection, mice were treated IP with vehicle control or CorA once a day (QD) at the indicated doses and treatment days. *Wolbachia ftsZ*/filarial actin per female worm was calculated 35 days post treatment onset. N = 3–5 mice with n = 7–48 worms per group. Statistical analysis was done using Kruskal-Wallis test with Dunn’s Multiple Comparisons post-hoc test. **** P<0.0001. Red lines indicate medians.

**Fig 2:**
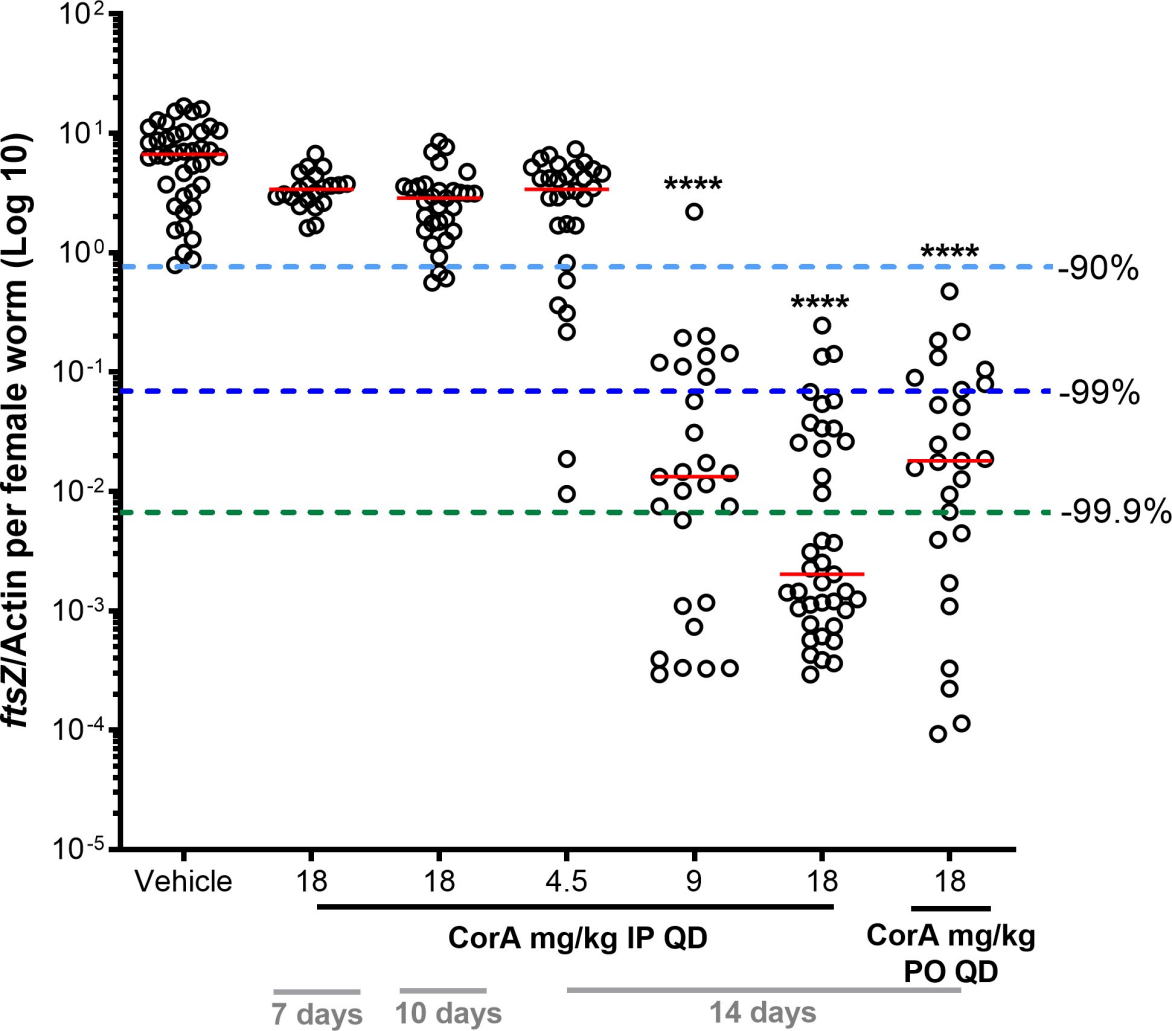
Fourteen-day CorA PO treatment depletes *Wolbachia* endosymbionts in the *L*. *sigmodontis* larval mouse model. One day after *L*. *sigmodontis* infection, mice were treated with vehicle control or CorA once a day (QD) at the indicated doses, treatment days and routes (PO or IP). *Wolbachia ftsZ*/filarial actin per female worm was calculated 35 days post treatment onset. N = 3–4 mice and n = 21–40 worms per group. Statistical analysis was done using Kruskal-Wallis test with Dunn’s Multiple Comparisons post-hoc test. **** P<0.0001. Red lines indicate medians.

The efficacy of CorA in mice infected with *L*. *sigmodontis* was determined by the *Wolbachia* reduction in female worms measured by *ftsZ*/actin compared to vehicle controls. Our *a priori* analysis calculated a need of at least 16 (experiment Fig 1) or 14 (experiment Fig 2) female worms to achieve a power >0.8. Based on the potential macrofilaricidal effect of CorA and our experience in regard to the worm burden of BALB/c mice and jirds infected with *L*. *sigmodonti*s, we used 3–5 mice (with an estimated 4–8 eligible worms per mouse) per group for the different studies.

The primary efficacy parameter of CorA in jirds infected with *L*. *sigmodontis* was the long-term clearance of MF from peripheral blood. Secondary efficacy parameters from jird experiments included the *Wolbachia* depletion from MF (*ftsZ*/MF) or adult female worms (*ftsZ*/worm) compared to the respective vehicle control group, embryograms from adult female worms from CorA treated jirds compared to the worms from the vehicle control group, as well as the median adult worm burden (jird experiments) compared between the treatment groups and the respective vehicle control group.

**Fig 3:**
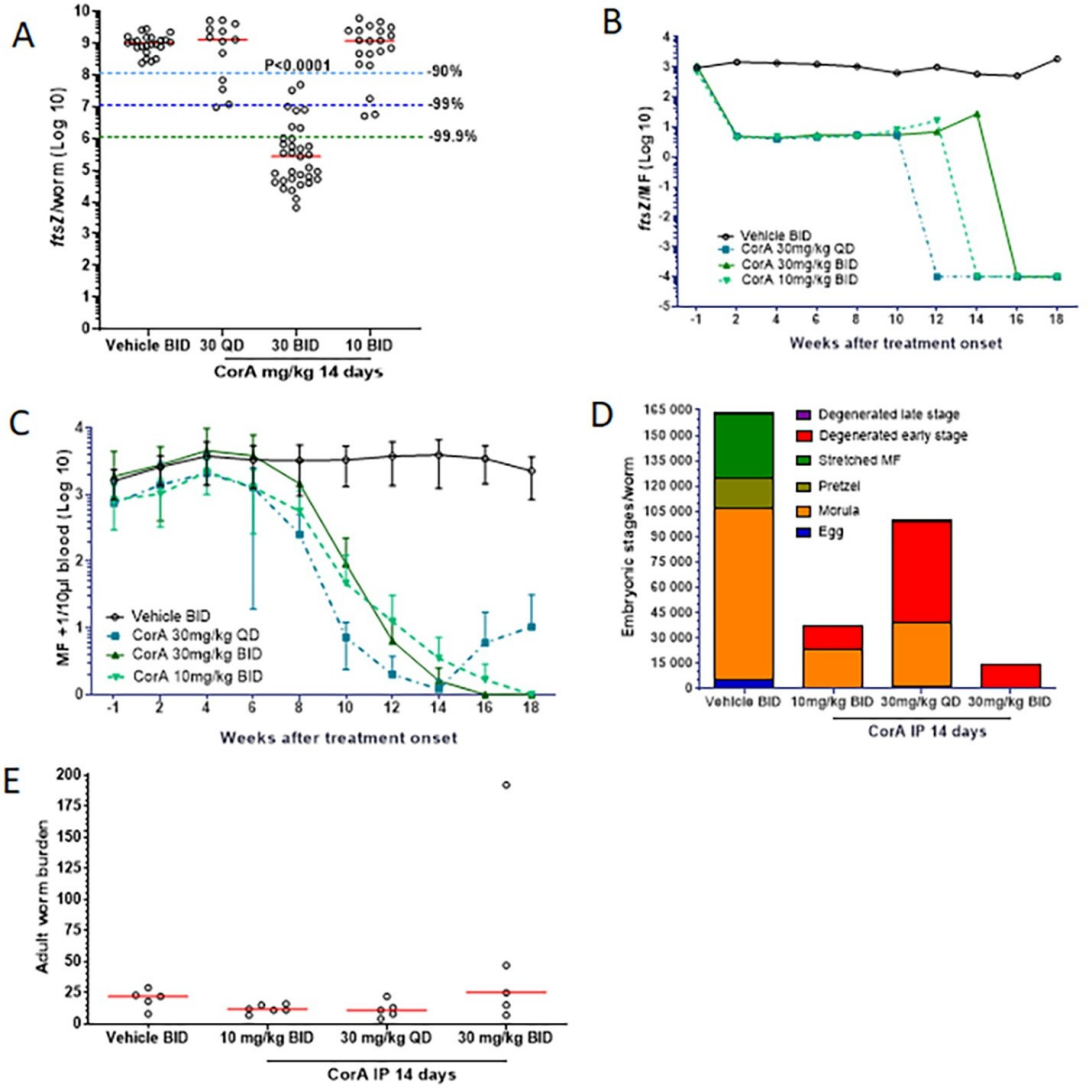
Fourteen-day CorA treatment depletes *Wolbachia* endosymbionts, clears microfilaremia and inhibits embryogenesis during chronic *L*. *sigmodontis* infection. Jirds patently infected with *L*. *sigmodontis* (12 weeks) were treated IP for 14 days with vehicle control, CorA 10 mg/kg BID, CorA 30 mg/kg QD, or CorA 30 mg/kg BID. Parasitological variables were monitored for 18 weeks, after which the worms were recovered for analysis of the efficacy of treatment. (A) *Wolbachia* in adult worms (median *ftsZ*/worm) and (B) kinetics of *Wolbachia* in MF (median *ftsZ*/MF) were quantified by qPCR. (C) Microfilaremia over time was expressed as MF+1/per 10 μL blood (mean±SD). (D) Embryograms assessed total eggs, morulae, pretzels, stretched MF, and degenerated early and late stages per female worm (mean from up to 5 worms/jird). (E) Adult worm burden 18 weeks after treatment onset. Representative figure from two experiments: 13–33 worms from n = 5–6 jirds per group (A), n = 5–6 jirds (B+C+E), n = 7–16 worms from n = 3–5 jirds per group (D). Statistical analysis was done using Kruskal-Wallis test with Dunn’s Multiple Comparisons post-hoc test (A). Red lines indicate medians.

**Fig 4:**
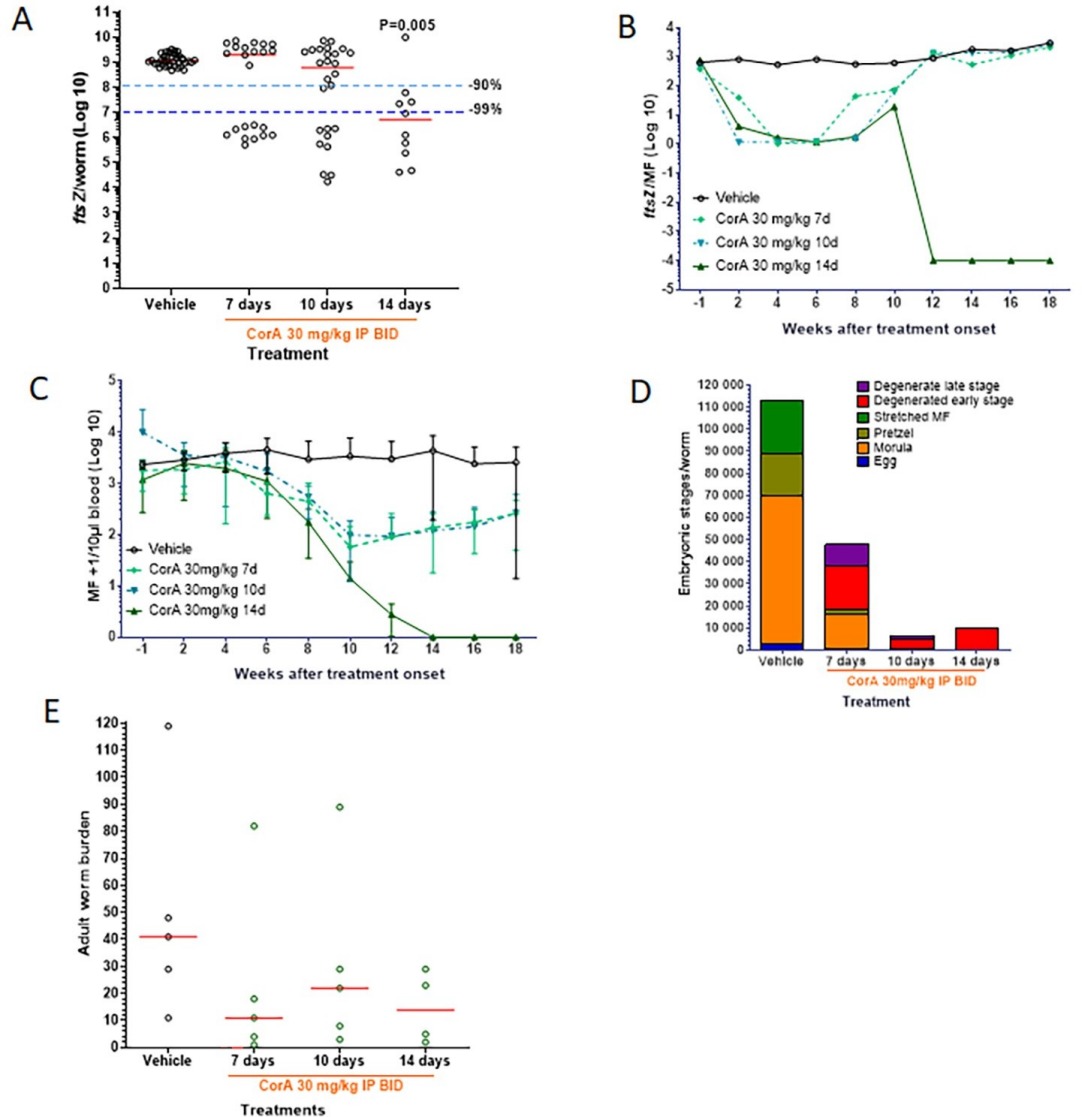
Fourteen-day CorA treatment is required for sustained clearance of microfilaremia and inhibition of embryogenesis. Jirds patently infected with *L*. *sigmodontis* (12 weeks) were treated IP with vehicle control for 14 days or CorA 30 mg/kg BID for 7, 10 or 14 days. Parasitological variables were monitored for 18 weeks, after which the worms were recovered for analysis. (A) *Wolbachia* in adult worms (median *ftsZ*/worm) and (B) kinetics of *Wolbachia* in MF (median *ftsZ*/MF) were quantified by qPCR. (C) Microfilaremia over time was expressed as MF+1/10 μL blood (mean±SD). (D) Embryograms assessed total eggs, morulae, pretzels, stretched MF, and degenerated early and late stages per female (mean from up to 5 worms/jird). (E) Adult worm burden 18 weeks after treatment onset. Representative figure from two experiments: 9–38 worms from n = 4–5 jirds per group (A), n = 4–5 jirds (B+C+E), n = 3–9 worms from n = 4–5 jirds per group (D). Statistical analysis was done using Kruskal-Wallis test with Dunn’s Multiple Comparisons post-hoc test. Red lines indicate medians.

**Fig 5:**
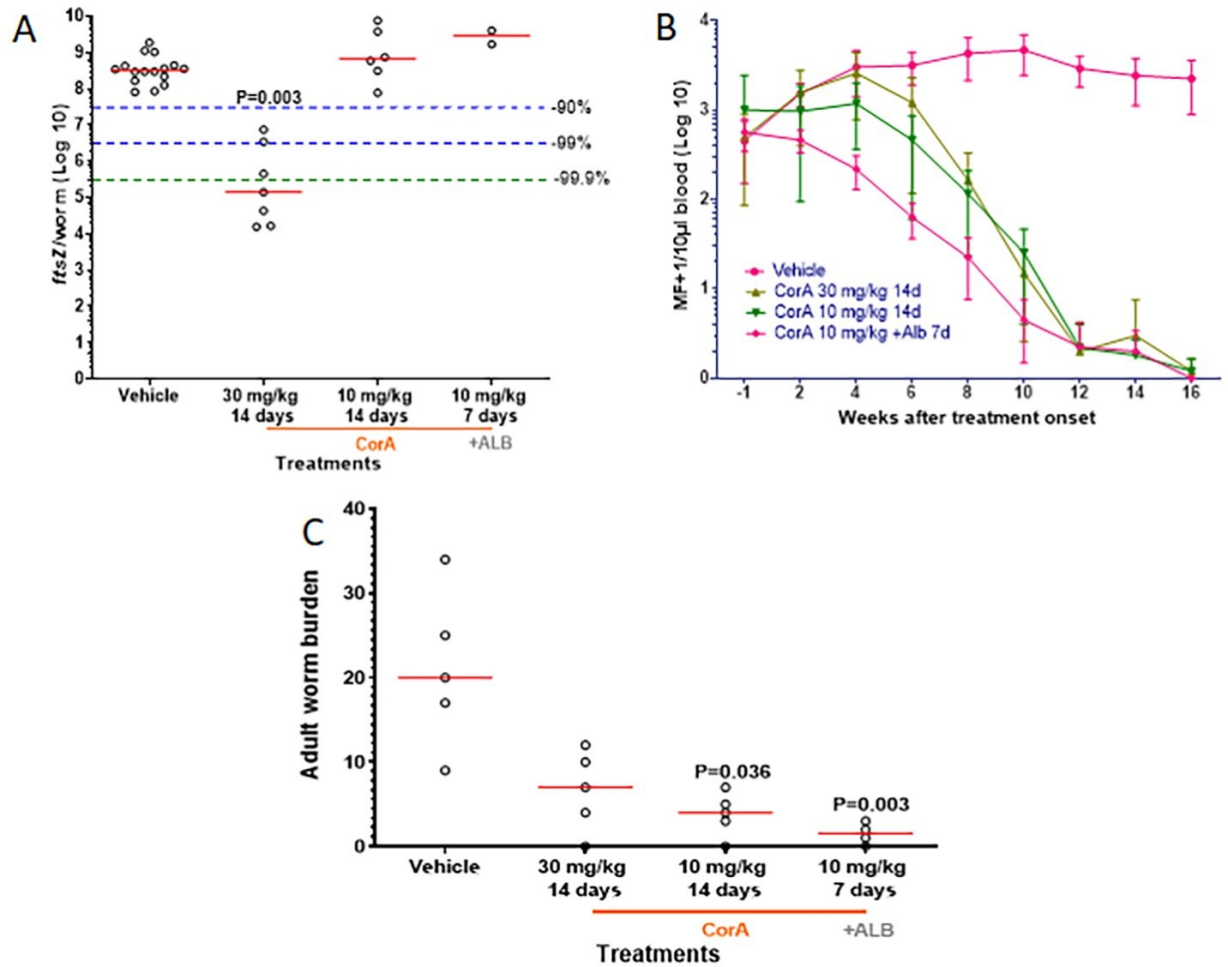
Seven-day CorA plus albendazole combination treatment clears microfilaremia and significantly reduces the adult worm burden. Jirds patently infected with *L*. *sigmodontis* (12 weeks) were treated IP with CorA 30 mg/kg, 10 mg/kg BID or 10 mg/kg plus PO 13 mg/kg albendazole (ALB) QD for 14 days, 14 days or 7 days, respectively. Parasitological variables were monitored for 16 weeks, after which the *L*. *sigmodontis* worms were recovered for analysis. (A) *Wolbachia* in adult worms (median *ftsZ*/worm) were quantified by qPCR. (B) Microfilaremia over time was expressed as MF+1/10 μL blood (mean±SD). (C) Adult worm burden 16 weeks after treatment onset. Each treatment group had 4–5 jirds and between 2–16 worms per group were available for analysis. Significant differences were calculated with the Kruskal-Wallis test with Dunn’s Multiple Comparisons post-hoc test. Red lines indicate medians.

For the jird studies (Figs 3–5) the primary endpoint was the reduction in MF. We therefore estimated a relatively large effect size. Thus, we calculated that 4–6 jirds per group would be needed to achieve a power >0.8.

Significant differences in *Wolbachia* content were calculated using the Kruskal-Wallis test with Dunn’s Multiple Comparisons post-hoc test comparing treatment groups to vehicle control using GraphPad Prism (Version 8.4 for Windows, GraphPad Software, San Diego, California USA, www.graphpad.com).

## Results

### Fourteen days of CorA treatment depletes *Wolbachia* endosymbionts

As a first approach to analyze the minimal dose and duration necessary for CorA anti-filarial efficacy, mice infected with *L*. *sigmodontis* L3 were treated IP 1 day after infection for 7, 10 or 14 days with different concentrations of CorA. All treatment regimens were well tolerated by the mice. *Wolbachia* reduction in female worms was evaluated at 35 dpi, at which time the *L*. *sigmodontis* have developed into adult filariae in untreated animals.

*Wolbachia* reduction was analyzed by quantifying copy numbers of the *Wolbachia ftsZ* gene per female worm. Seven-day CorA treatment resulted in minor reductions in *Wolbachia* (Fig 1), while the 10-day regimens significantly depleted *Wolbachia*. The 18 or 36 mg/kg CorA regimens depleted *Wolbachia* by more than 90% (18 mg/kg: 94%, P≤0.0001; 36 mg/kg: 98%, P<0.0001). Administering 9 mg/kg CorA for 14 days reduced *Wolbachia* by 98.3% (P<0.0001) and 18 mg/kg CorA treatment resulted in a reduction of 99.96% (P<0.0001). The latter dose achieved depletion of *Wolbachia* by >99% in all worms analyzed, and by >99.9% in the majority of worms analyzed.

Based on the target product profile (TPP) of new macrofilaricidal drug candidates (www.dndi.org/wp-content/uploads/2018/05/Monnot_TPPOnchocerciasis_UgandaMeeting2018.pdf), oral administration is preferred. Therefore, PO CorA treatment was compared to IP regimens. Treatment of mice with 18 mg/kg CorA PO for 14 days depleted *Wolbachia* by 99.7% (P = 0.002) and was comparable to IP treatment with 9 mg/kg CorA for 14 days (*Wolbachia* reduction: 99.8%, P≤0.0001) (Fig 2).

### Fourteen-day CorA treatment depletes *Wolbachia* endosymbionts, clears microfilaremia and inhibits embryogenesis during chronic filarial infection

As Mongolian gerbils (jirds) have an increased susceptibility for infection with *L*. *sigmodontis* in comparison to mice and allow infections exceeding 6 months [36], we used the *L*. *sigmodontis* jird model to analyze the efficacy of CorA treatment during patent filarial infections (approx. 12 weeks after infection). Treatment was only initiated in MF-positive jirds.

In the first jird experiment, the impact of different CorA dosages administered for 14 days IP was evaluated. Treatment with 10 mg/kg CorA BID or 30 mg/kg CorA QD had no impact on the *Wolbachia* loads in adult female worms recovered at 18 weeks post treatment onset (wpt; Fig 3A). However, treatment with 30 mg/kg BID reduced *Wolbachia* by >99.9%. Although 10 mg/kg BID and 30 mg/kg QD treatment had no long-term impact on the *Wolbachia* load in adult female worms, *Wolbachia* in MF were reduced in all groups starting 2 wpt (Fig 3B). After 12–16 wpt, no MF were present in the CorA treatment groups for *Wolbachia* analysis (indicated by setting *ftsZ*/MF to a value of 10^−4^). In parallel, a reduction in MF was seen at 8 wpt and reached 0 at 14, 16 and 18 wpt in animals treated for 14 days with 30 mg/kg QD, 30 mg/kg BID, and 10 mg/kg BID CorA, respectively (Fig 3C). In all CorA groups treated BID for 14 days, all animals became amicrofilaremic. However, MF rebounded in 1/5 animals at 16 wpt in the 30 mg/kg QD treated group.

To test whether CorA treatment inhibits filarial embryogenesis in adult female worms, embryograms of female worms not used for qPCR were generated and the different embryonic stages counted (Fig 3D). In the vehicle control group all normal developed embryonic stages (eggs, morula, pretzel and stretched MF) were found and female worms harbored almost no degenerated stages. All CorA treatment regimens reduced the number of embryonic stages per female worm and, apart from the morula stage, the majority of recovered embryonic stages were degenerated early stages (eggs and morulae). Treatment with 30 mg/kg CorA BID inhibited embryogenesis the most and resulted in mainly degenerated early stages with a few degenerated late stages and morula stages in only 1/16 analyzed worms.

The number of adult worms from each animal were counted. CorA treatments of 10 mg/kg BID and 30 mg/kg QD for 14 days reduced the worm burden, while CorA 30 mg/kg BID treatment given for 14 days did not.

In the second jird experiment, 30 mg/kg given 14 days was compared to 20 mg/kg given for 14 or 10 days (S2 Fig). Doses of 20 mg/kg CorA given BID for 14 days reduced *Wolbachia* by 98%, but the same dose given for 10 days resulted in apparent rebound in adult female worms (S2A Fig) and MF (S2B Fig). A reduction in MF was seen at 8 wpt and reached 0 at 16 wpt in animals treated for 14 days with 20 mg/kg BID and 30 mg/kg BID (S2C Fig). CorA treatment at 20mg/kg BID for 10 days was not sufficient to completely clear the microfilaremia (S2C Fig). Embryograms were performed on available worms. The vehicle control group contained normal developing embryonic stages (eggs, morula, pretzel and stretched MF) and harbored almost no degenerated stages (S2D Fig). All CorA treatment regimens reduced the number of embryonic stages per female worm and the majority of recovered embryonic stages were degenerated early stages (eggs and morulae). Both 14-day 20 mg/kg BID and 30 mg/kg BID treatments reduced the worm burden (S2E Fig).

### Fourteen-day CorA treatment is required for sustained clearance of microfilaremia and inhibition of embryogenesis

Having established that 30 mg/kg CorA BID treatment clears *Wolbachia* endosymbionts that inhibits embryogenesis and results in sustained amicrofilaremia during chronic *L*. *sigmodontis* infection, we assessed in the third jird study whether shorter 30 mg/kg CorA BID treatment regimens of 7 or 10 days are efficacious. Similar to the previous experiments (Fig 3, S2 Fig), 14-day CorA BID IP treatment at 30 mg/kg significantly reduced *Wolbachia* loads in adult female worms by 99.55% (P<0.01) at 18 wpt (Fig 4A). Shorter treatment duration of 10 days resulted in a >2-log drop of *Wolbachia* in 9/26 worms analyzed, while the remainder did not show significant *Wolbachia* reduction compared to worms from control animals, indicating borderline efficacy and a rebound of *Wolbachia* at this dose, as observed earlier with other anti-wolbachials [32]. A similar result was observed with worms from 7-day CorA-treated animals (Fig 4A).

*Wolbachia* loads in MF started to decline in all CorA treatment groups after 2 wpt. While the 7-day and 10-day treatment regimens resulted in a rebound of *Wolbachia* commencing at 8–10 wpt (Fig 4B), the 14-day CorA regimen achieved complete clearance of MF 14 wpt and therefore prevented the analysis of *Wolbachia*/MF in this group at later time points (Fig 4C). MF counts in all CorA-only treatment groups started to decline 8 wpt, but animals from the 7-day and 10-day CorA treatment regimens showed a rebound in MF counts starting at 12 wpt (Fig 4C).

Seven-day, 10-day and 14-day regimens with 30 mg/kg CorA BID reduced the total embryonal stages (Fig 4D). The 10-day and 14-day CorA treatments resulted in the recovery of mostly degenerated early stages (eggs, morulae) from female worms, while the 7-day CorA treatment did not completely inhibit the formation of morulae and pretzel stages.

Similar to the results shown in Fig 3E and S2E Fig, CorA-only treatments given for 7, 10 or 14 days reduced the adult worm recovery (Fig 4E).

### Seven-day CorA plus albendazole combination treatment clears microfilaremia and significantly reduces the adult worm burden

As a recent publication has shown synergy of ALB in combination with other anti-wolbachial drugs [37], we examined in the fourth jird experiment whether the combination of CorA and ALB treatment could allow shorter treatment times and lower doses of CorA. The infected jirds were administered a 7-day treatment IP regimen of 10 mg/kg CorA BID beginning 12 wpi, with ALB administered PO 30 minutes after CorA administration. Additional animals were treated IP for 14 days with vehicle control, CorA 30 mg/kg BID or CorA 10 mg/kg BID.

Confirming the results shown in Figs 3, 4 and S2 Fig, CorA administered at 30 mg/kg BID, but not 10 mg/kg BID, for 14 days significantly depleted the *Wolbachia* from the adult worms (P = 0.015, Fig 5A). No reduction in *Wolbachia* was observed in the two female worms available for qPCR analysis from animals treated with the CorA-ALB combination for 7 days (Fig 5A).

Animals treated for 7 days BID with 10 mg/kg CorA plus ALB showed an earlier reduction in the MF counts, with >50% MF cleared at 4 wpt compared to 8 wpt with the 14-day CorA-only regimens. Both the 14-day CorA-only treatments as well as the 7-day combination treatment resulted in amicrofilaremia in all animals studied at 16 wpt (Fig 5B).

Similar to the previous results, 14-day CorA-only treatment regimens reduced the adult worm burden significantly with 10 mg/kg BID and by trend with 30 mg/kg BID (Fig 5C). In total, 3 out of 4 experiments using the 14-day BID CorA-only treatment with 30 mg/kg and 2 out of 2 experiments using the 14-day BID CorA-only treatment with 10 mg/kg had macrofilaricidal efficacy. Administration of CorA at 10 mg/kg plus ALB for 7 days resulted in the strongest reduction of the adult worm burden by 95% (P = 0.003, Fig 5C). We have demonstrated earlier that, on its own, ALB does not show anti-filarial effects when administered for 7 days [41]. In the experiment displayed in Fig 4, we had CorA 30 mg/kg BID plus ALB as an additional 7-day treatment arm, which had a significant reduction of the adult worm burden by 93% (P<0.01).

## Discussion

The present study confirms CorA as a promising candidate for the treatment of filarial diseases. Two weeks of *in vivo* CorA treatment were sufficient to deplete *Wolbachia* endosymbionts in the *L*. *sigmodontis* model, that resulted in sustained MF clearance, inhibition of embryogenesis, and macrofilaricidal efficacy during chronic filarial infection. Combination therapy of CorA and ALB further enhanced the macrofilaricidal efficacy with a shortened treatment regimen of 7 days and a CorA dose as low as 10 mg/kg.

Depletion of *Wolbachia* endosymbionts from filariae by doxycycline therapy was previously shown to inhibit filarial development and to mediate the clearance of MF and permanent inhibition of filarial embryogenesis in patients with onchocerciasis, lymphatic filariasis or mansonelliasis [22,42–47], leading to a slow and well-tolerated killing of the adult filariae after 18 months in lymphatic filariasis [48,49] (ISRCTN15216778), and later in onchocerciasis [50,51]. However, while making it the first safe macrofilaricidal drug for onchocerciasis, the long 4-5-week treatment regimen with doxycycline and its contraindications limit its broader use beyond individual prescriptions. Importantly, a shorter regimen with doxycycline at human equivalent dose in the *L*. *sigmodontis* model in jirds confirmed human studies that determined that doxycycline administered for 2 weeks is insufficient to deplete *Wolbachia* or result in long-term sterilizing or macrofilarididal effects [32], thus further validating the predictive power of the *L*. *sigmodontis* model.

Novel drug candidates with an improved efficacy against the *Wolbachia* endosymbionts were recently investigated in preclinical studies with the *L*. *sigmodontis* rodent model and additional models of filariasis (Table 1) [29,31–35,41,52]. While those drug candidates also achieved shortened treatment regimens of 7–14 days to deplete *Wolbachia* endosymbionts in the *L*. *sigmodontis* model, the CorA-only and CorA plus ALB therapies are the first anti-wolbachial treatment published in the *L*. *sigmodontis* model that have achieved robust macrofilaricidal efficacy. This indicates that the clearance of the adult worms by CorA (plus ALB) treatment occurs within a few months after treatment.

**Table 1:** Comparison of current anti-wolbachial pre-clinical candidates for macrofilaricidal efficacy in *Meriones unguiculatus*.

Compound	Infection, Treatment time	Comments
ABBV-4083 [32,53]	*Litomosoides sigmodontis*, 14 days QD	No published macrofilaricidal activity
AWZ1066 [34]	*Brugia malayi* and *L*. *sigmodontis*, 7 days BID	No published macrofilaricidal activity
Corallopyronin A	*L*. *sigmodontis* infection in Mongolian gerbils, 14 days BID	Macrofilaricidal
	*L*. *sigmodontis* infection in Mongolian gerbils, 7 days BID plus 7 days Albendazole QD	
Doxycycline [30,32]	*L*. *sigmodontis* 2 weeks BID	Sub-optimal, rebound of *Wolbachia* and MF
Rifampicin [54]	*Brugia pahangi*, 7 days BID	Sub-optimal, rebound of *Wolbachia* and MF

Although we did not directly compare efficacy of the combination of CorA plus ALB to doxycycline plus ALB or doxycycline plus rifampicin, there is evidence that combining a tetracycline with ALB does not result in shorter treatment times than 4 weeks. In the jird *Brugia* model, treating infected animals with a combination of minocycline, which is more effective than doxycycline [55], for 15 days in combination with ALB for 3 days resulted in ≤ 1-log drop (90%) reduction in *Wolbachia* [37]. A 1-log reduction in *Wolbachia* is insufficient to result in anti-filarial activity seen with a 4-week regimen of doxycycline. With these experiments in mind, we refrained from adding a doxycycline plus ALB group in these experiments, as we considered it very unlikely that the effect would be equivalent to CorA 7-days plus ALB. However, we will perform this direct comparison with our CorA oral formulation that is under development.

With regard to the different doses tested in the jird and mouse model, the PK in jirds and mice differs and jirds require higher doses. The intrinsic clearance of CorA is 9-10X higher in liver microsomes from rats compared to microsomes from humans or mice. There are no data for Mongolian gerbils, but if they are similar to rats, then the intrinsic clearance rate would also be high in this species and require a BID regimen in jirds to achieve efficacy.

BID treatment in jirds will not necessarily translate into BID for human regimens. Furthermore, the experiments done in mice were shorter and did not allow the investigation of a rebound in the *Wolbachia*, as this starts to occur around 14 weeks post treatment start. Therefore, low CorA doses shown to deplete *Wolbachia* in mice may lead to insufficient *Wolbachia* clearance and therefore possible rebound of *Wolbachia* in the *L*. *sigmodontis* jird model.

A limitation of our study is the usage of ALB as a 7-day regimen, while within MDA the drug is usually used as a single dose. In the *Brugia malayi* preclinical model of filariasis, although ALB treatment synergized with anti-wolbachials and led to the clearance of *Wolbachia* endosymbionts after 7 days in combination with rifampicin [37], or in a regimen of 15 days of minocycline plus 3 days of ALB [50], the reduction in *Wolbachia* was <1 log and therefore did not result in block of embryogenesis and loss of MF. Future studies are needed to determine whether shorter regimens of CorA can synergize with ALB given for ≤3 days to achieve macrofilaricidal efficacy. On its own, ALB does not show anti-filarial effects when administered for 7 days [41].

A second limitation of our study is that although the jird experiments allowed the investigation of CorA efficacy as a macrofilaricidal compound, because the parasites live long enough to underpin drug efficacy, we administered CorA IP rather than PO. We choose this because the oral formulation for CorA is still being developed in parallel to the efficacy experiments. Therefore, we administered CorA IP to avoid issues of possible suboptimal oral bioavailability in the jird experiments. Additionally, the current vehicle contains 50% PEG400, which can cause diarrhea in animals when given for many days and IP administration avoided this source of possible stress to the animals. For usage in humans, an oral formulation is being developed and this will determine the final dosing regimen.

In principle, another limitation of our study is the fact that we used a rodent filarial nematode as model for human filariasis with a comparatively long, albeit still limited, observation period of ≥16 wpt. However, the *L*. *sigmodontis* model has been used to demonstrate first efficacy of doxycycline against filariae by depletion of *Wolbachia* endosymbionts [56], which was subsequently confirmed by clinical studies in onchocerciasis and lymphatic filariasis patients [22,23,57,58]. Furthermore, the *L*. *sigmodontis* model has been used by the Macrofilaricidal Drug Accelerator program, the Bill & Melinda Gates Foundation and the Drugs for Neglected Diseases *initiative* (DND*i*) to identify novel drug candidates for onchocerciasis (Table 1) [30,32–35,59]. These studies identified several novel candidates, including the anti-wolbachial candidates ABBV-4083 (in preparation for phase 2 clinical studies) and AWZ1066S (pre-clinical development for phase 1 clinical studies) [30,32,34,53].

CorA is being developed to clinical phase 1 within the German Center for Infection Research (DZIF, https://www.dzif.de/en/location/bonn-koln) and the European Commision Horizon 2020 funded Helminth Elimination Platform (HELP, https://eliminateworms.org/). As part of this development, a hurdle to production of the natural product has been overcome by transferring the gene cluster into *Myxococcus xanthus*, which is conducive to industrial-scale production [39]. Additionally, the purification of CorA has been simplified by removal of preparative HPLC (high-pressure liquid chromatography) and achieving 92–98% purity. Preclinical ADMET (absorption, distribution, metabolism, excretion and toxicity) has not resulted in any relevant issues in the continued development of CorA [60] (results will be published separately). Consistently, we have never observed side effects of CorA treatment in any of the animal species and dosing regimens used to demonstrate preclinical proof-of-concept. Using the FDA conversion of animal dose to Human Equivalent Dose based on body surface area (HED = animal dose in mg/kg x (animal weight in kg/human weight in kg)^0.33^ [61]), the macrofilaricidal dose of 10 mg/kg CorA plus ALB equals a human dose of 1.4 mg/kg CorA.

Although not the focus of this manuscript, CorA also has efficacy against other Gram-negative intracellular bacteria that cause diseases in humans, *Rickettsia spp*., *Orientia tsutsugamushi*, and *Chlamydia trachomatis* [62,63]. CorA efficacy against rifampicin-resistant *S*. *aureus* and MRSA was published when CorA was first discovered and characterized [26,64]. Our preclinical development plan has been made so that the data generated will allow for registration of CorA for use against human filarial infections and also allow expanding the registration to these other infections.

Based on the above described CorA efficacy, its improved production and excellent pharmacological and safety profiles, CorA is a promising anti-*Wolbachia* drug candidate with reduced treatment times to replace doxycycline for the treatment of filariasis [19], especially in end game activities and post-MDA. CorA alone, but particularly in combination with ALB treatment, results in robust macrofilaricidal efficacy and thus has a performance well within the ideal TPP for a new macrofilaricidal drug against onchocerciasis (www.dndi.org/wp-content/uploads/2018/05/Monnot_TPPOnchocerciasis_UgandaMeeting2018.pdf).

### Material & correspondence

Please address correspondence and material requests to AH and KP.

## Supporting information

S1 Fig 1H NMR spectrum (300 MHz) of Corallopyronin A in dimethyl sulfoxide-*d_6_* with the internal reference compound dimethyl sulfone. (PDF)

S2 Fig Fourteen-day CorA treatment with 30 and 20 mg/kg depletes *Wolbachia* endosymbionts, clears microfilaremia and inhibits embryogenesis during chronic *L. sigmodontis* infection. Jirds patently infected with *L*. *sigmodontis* (15 weeks) were treated BID with CorA 30 mg/kg for 14 days, or CorA 20 mg/kg for 10 or 14 days. Parasitological variables were monitored for 21 weeks, after which the *L*. *sigmodontis* worms were recovered for analysis of the efficacy of treatment. (A) *Wolbachia* (median *ftsZ*/worm) in adult worms and (B) kinetics of *Wolbachia* depletion in microfilariae (median *ftsZ*/MF) were quantified by qPCR. (C) Microfilaremia over time was expressed as MF+1/10 μL blood (mean±SD). (D) Embryograms assessed total eggs, morulae, pretzels, stretched MF, and degenerated early and late stages per female worm (median from up to 5 worms/jird). (E) Adult worm burden at 21 weeks after treatment onset. Each treatment group had 4–9 jirds and between 13–33 worms per group were available for analysis. Significant differences were calculated with the Kruskal-Wallis test with Dunn’s Multiple Comparisons post-hoc test GraphPad Prism (Version 8.4 for Windows, GraphPad Software, San Diego, California USA, www.graphpad.com). Red line indicates medians.
(PDF)
