# Supplementary material for: Corallopyronin A for short-course anti-wolbachial, macrofilaricidal treatment of filarial infections
Source: PLoS Negl Trop Dis. 2020 Dec 7;14(12):e0008930. doi: 10.1371/journal.pntd.0008930 (PMC7746275; doi:10.1371/journal.pntd.0008930)
Supplement: S1 Fig — (PDF) [file pntd.0008930.s001.pdf]

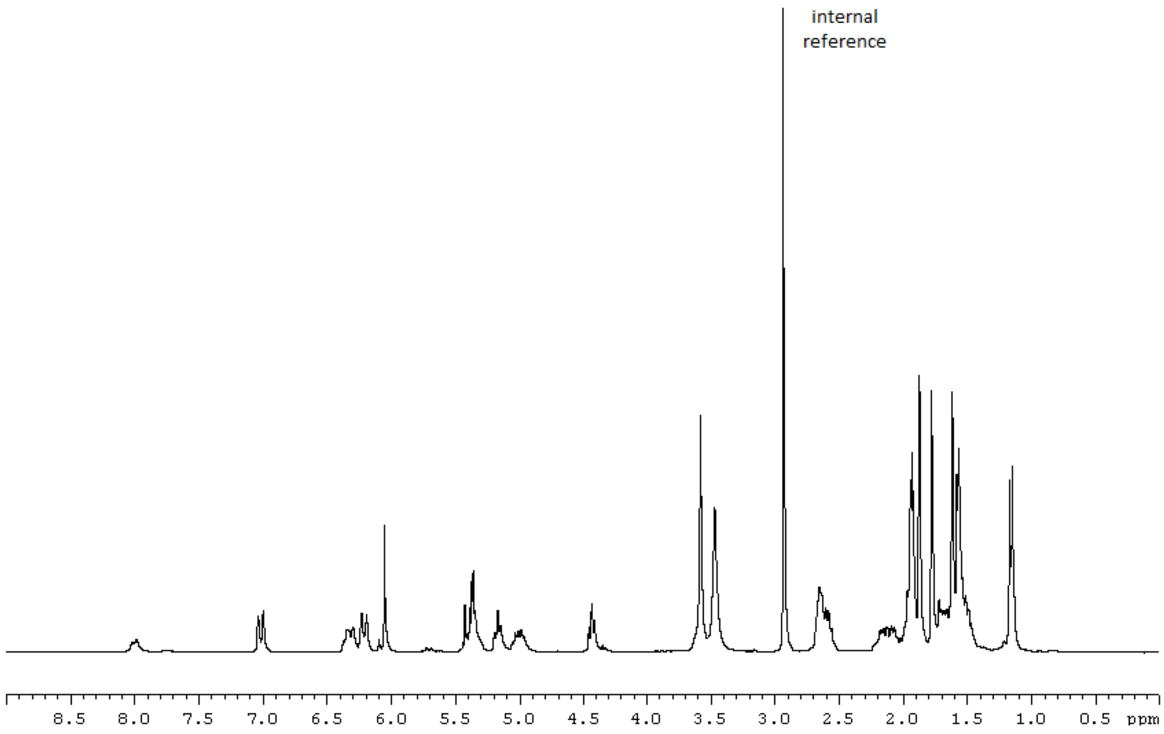

S1 Fig. ^{1}H NMR spectrum (300 MHz) of Corallopyronin A in dimethyl sulfoxide-*d_6* with the internal reference compound dimethyl sulfone.

Supplementary Method

Thirty mg of CorA and 4 mg of the reference compound dimethyl sulfone (99.96 % purity; Sigma-Aldrich, catalog no.: 41867) were weighed into 2 mL glass vials. Then 0.7 mL of dimethyl sulfoxide-d_6 (≥ 99.8 atom % D; e.g., Deutero GmbH, order-no.: 00905-075) were added and mixed thoroughly with a vortexer and ultrasonic water bath at room temperature. The solution was then transferred to a 5 mm NRM tube for measurement using the following experimental parameters:

**Instrument:**	Bruker Avance DPX300; Topspin 1.3 software
**Detection:**	^{1}H
**Solvent:**	Dimethyl sulfoxide-d_6
**Shift reference:**	Singlet of reference compound at δ 2.99
**Spectrometer frequency:**	300 MHz
**Exciting pulse:**	30°
**Spin rotation:**	without
**Sample temperature:**	303 K

Relaxation delay: 30 s
Acquisition time: ≥ 4 s
Sweep width: 16 ppm
Time domain points: ≥ 64 k
Exciting frequency (O1): 5 ppm
Number of scans: ≥ 100
Line broadening: 0.3 Hz
Spectrum data points: ≥ 64 k

The sample was shimmed so that the width at half maximum of the singlet resonance of the reference compound (δ 2.99) was less than 2 Hz.

Processing, evaluation, and calculation

To quantify the content of CorA, the integral of the singlet resonance at δ 6.06 (CH; 1H) was compared with the integral of the singlet resonance of the reference compound at δ 2.99 (2 x CH_3; 6H). For the determination of the left and right limits of integration the width at half maximum (in Hz) of both resonances were multiplied by the factor 12 and the resulting value subsequently added (for the left limit) and subtracted (for the right limit), starting from the middle of the resonance, respectively. The content was calculated according to the following equation:

$$P_x = \frac{I_x}{I_{Std}} \cdot \frac{no_{Std}}{no_X} \cdot \frac{M_x}{M_{Std}} \cdot \frac{m_{Std}}{m_x} \cdot P_{Std}$$

P = content in %; m = sample weight; M = molecular weight (dimethyl sulfone: 94.13 g/mol; CorA: 527.65 g/mol); I = signal intensity; no = number of protons (dimethyl sulfone; δ 2.99, 6H; CorA: δ 6.06, 1H); x = CorA; Std = dimethyl sulfone
