# Supplementary material for: Corallopyronin A for short-course anti-wolbachial, macrofilaricidal treatment of filarial infections
Source: PLoS Negl Trop Dis. 2020 Dec 7;14(12):e0008930. doi: 10.1371/journal.pntd.0008930 (PMC7746275; doi:10.1371/journal.pntd.0008930)
Supplement: S2 Fig — Jirds patently infected with L. sigmodontis (15 weeks) were treated BID with CorA 30 mg/kg for 14 days, or CorA 20 mg/kg for 10 or 14 days. Parasitological variables were monitored for 21 weeks, after which the L. sigmodontis worms were recovered for analysis of the efficacy of treatment. (A) Wolbachia (median ftsZ/worm) in adult worms and (B) kinetics of Wolbachia depletion in microfilariae (median ftsZ/MF) were quantified by qPCR. (C) Microfilaremia over time was expressed as MF+1/10 μL blood (mean±SD). (D) Embryograms assessed total eggs, morulae, pretzels, stretched MF, and degenerated early and late stages per female worm (median from up to 5 worms/jird). (E) Adult worm burden at 21 weeks after treatment onset. Each treatment group had 4–9 jirds and between 13–33 worms per group were available for analysis. Significant differences were calculated with the Kruskal-Wallis test with Dunn’s Multiple Comparisons post-hoc test GraphPad Prism (Version 8.4 for Windows, GraphPad Software, San Diego, California USA, www.graphpad.com). Red line indicates medians. (PDF) [file pntd.0008930.s002.pdf]

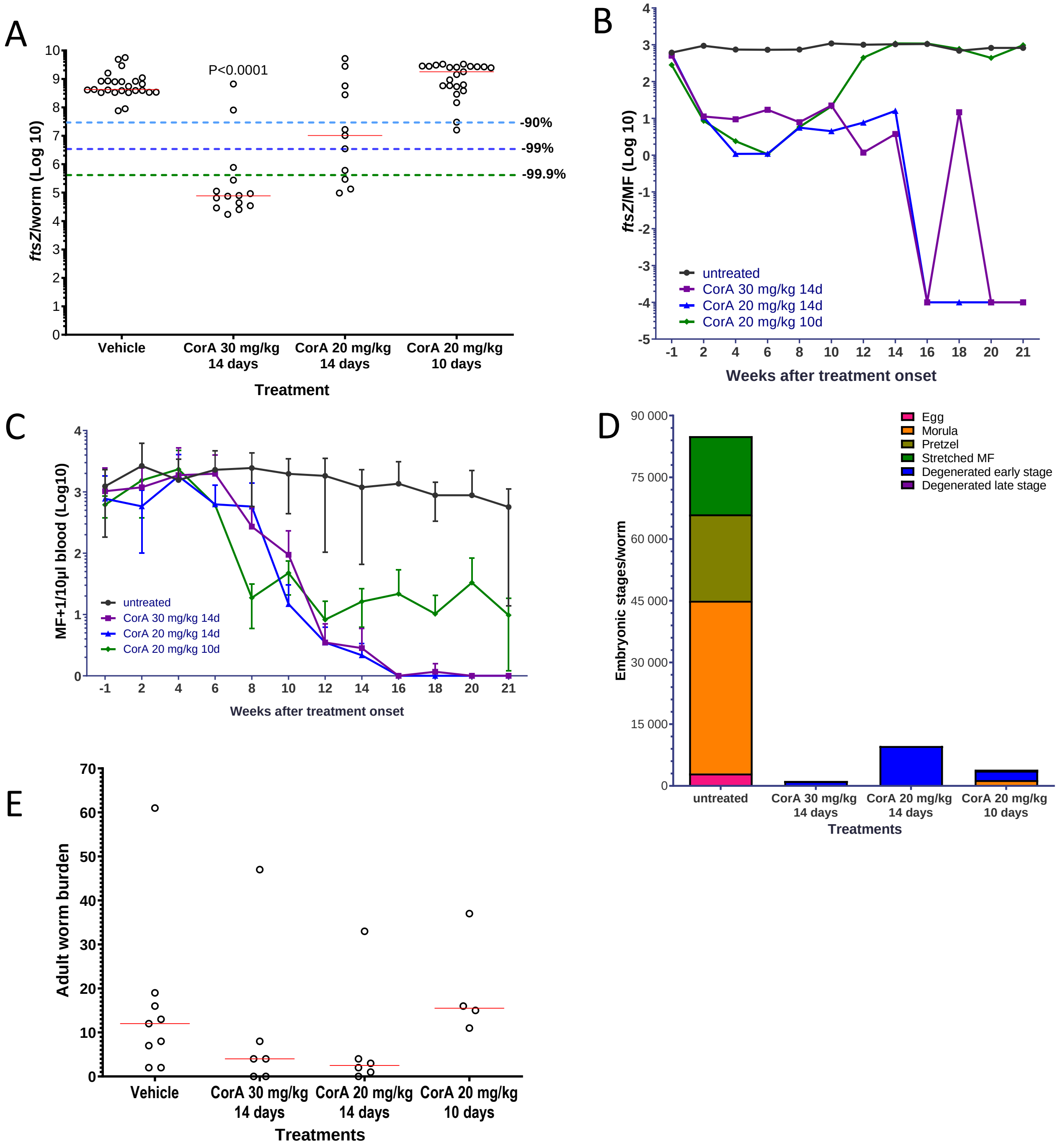

S2 Fig. Fourteen-day CorA treatment with 30 and 20 mg/kg depletes *Wolbachia* endosymbionts, clears microfilaremia and inhibits embryogenesis during chronic *L. sigmodontis* infection. Jirds patently infected with *L. sigmodontis* (15 weeks) were treated BID with CorA 30 mg/kg for 14 days, or CorA 20 mg/kg for 10 or 14 days. Parasitological variables were monitored for 21 weeks, after which the *L. sigmodontis* worms were recovered for analysis of the efficacy of treatment. (A) *Wolbachia* (median *ftsZ*/worm) in adult worms and (B) kinetics of *Wolbachia* depletion in microfilariae (median *ftsZ*/MF) were quantified by qPCR. (C) Microfilaremia over time was expressed as MF+1/10 µL blood (mean±SD). (D) Embryograms assessed total eggs, morulae, pretzels, stretch MF, and degenerated early and late stages per female worm (median from up to 5 worms/jird). (E) Adult worm burden at 21 weeks after treatment onset. Each treatment group had 4-9 jirds and between 13-33 worms per group were available for analysis. Significant differences were calculated with the Kruskal-Wallis test with Dunn's Multiple Comparisons post-hoc test GraphPad Prism (Version 8.4 for Windows, GraphPad Software, San Diego, California USA, www.graphpad.com). Red line indicates medians.
